# The possible impact of levosimendan infusions on clinical course of heart failure: the results of the LEIA-HF study

**DOI:** 10.1093/eschf/xvag021

**Published:** 2026-01-16

**Authors:** Ewelina Kazimierczyk, Remigiusz Kazimierczyk, Anna M Imiela, Dominika Południewska, Ewelina Rogalska, Anna Szyszkowska, Klaudia Mickiewicz, Franciszek Kukliński, Teresa Fernandez-Moreno, Marek Gierlotka, Ewa Straburzyńska-Migaj, Jadwiga M Nessler, Agnieszka Pawlak, Mariusz Gąsior, Jacek Kubica, Miłosz Jaguszewski, Jarosław D Kasprzak, Agata Bielecka-Dąbrowa, Wojciech Wojakowski, Przemysław Leszek, Łukasz Szarpak, Agnieszka Tycińska

**Affiliations:** Department of Cardiology and Internal Medicine with Cardiac Intensive Care Unit, Medical University of Bialystok, Bialystok, Poland; Department of Cardiology and Internal Medicine with Cardiac Intensive Care Unit, Medical University of Bialystok, Bialystok, Poland; Department of Internal Disease and Cardiology, Center for Management of Venous Thromboembolic Disease, Medical University of Warsaw, Warsaw, Poland; Department of Cardiology and Internal Medicine with Cardiac Intensive Care Unit, Medical University of Bialystok, Bialystok, Poland; Department of Cardiology and Internal Medicine with Cardiac Intensive Care Unit, Medical University of Bialystok, Bialystok, Poland; Department of Cardiology and Internal Medicine with Cardiac Intensive Care Unit, Medical University of Bialystok, Bialystok, Poland; Department of Cardiology and Internal Medicine with Cardiac Intensive Care Unit, Medical University of Bialystok, Bialystok, Poland; Department of Intensive Cardiac Care, Medical University of Bialystok, Bialystok, Poland; Department of Intensive Cardiac Care, Medical University of Bialystok, Bialystok, Poland; Department of Cardiology, Institute of Medical Sciences, University of Opole, Opole, Poland; First Department of Cardiology, Poznan University of Medical Sciences, Poznan, Poland; Department of Coronary Disease and Heart Failure, Institute of Cardiology, Jagiellonian University Medical College, Krakow, Poland; Department of Cardiology, National Medical Institute of the Ministry of Interior and Administration, Warszawa, Poland; 3rd Department of Cardiology, Faculty of Medical Sciences in Zabrze, Medical University of Silesia, Katowice, Poland; Department of Cardiology and Internal Medicine, Collegium Medicum in Bydgoszcz, Nicolaus Copernicus University in Torun, Bydgoszcz, Poland; First Department of Cardiology, Medical University of Gdansk, Gdansk, Poland; First Department of Cardiology, Medical University of Lodz, Lodz, Poland; Department of Cardiology and Adult Congenital Diseases, Polish Mother's Memorial Hospital Research Institute (PMMHRI), Lodz, Poland; Department of Preventive Cardiology and Lipidology, Medical University of Lodz, Lodz, Poland; Division of Cardiology and Structural Heart Diseases, Medical University of Silesia, Katowice, Poland; Department of Heart Failure and Transplantology, National Institute of Cardiology, Warsaw, Poland; Institute of Medical Sciences, The John Paul II Catholic University of Lublin, Lublin, Poland; Department of Clinical Research and Development, LUXMED Group, Warsaw, Poland; Department of Cardiology and Internal Medicine with Cardiac Intensive Care Unit, Medical University of Bialystok, Bialystok, Poland; Department of Intensive Cardiac Care, Medical University of Bialystok, Bialystok, Poland

**Keywords:** Congestion, Heart failure, Inotropes, Levosimendan

## Abstract

**Introduction:**

Intermittent administration of levosimendan has recently been introduced for long-term use in patients with advanced heart failure (HF). However, the impact of this therapy on survival remains inconclusive.

**Methods:**

Levosimendan in ambulatory HF patients was a multicentre, randomized, double-blind, placebo-controlled, Phase IV clinical trial of intermittent levosimendan administration in patients with ambulatory stable advanced HF [left ventricular ejection fraction ≤35%, New York Heart Association Classes III and IV]. The primary clinical endpoint of the study was composed of death from any cause or unplanned hospitalization for HF, whichever occurred first in a 12-month follow-up period. Infusion started at a dose of 0.05 μg/kg/min and lasted ∼24 h (up to a maximum dose of 12.5 mg) every 4 weeks. The study was conducted in nine centres around Poland. The study was prematurely terminated due to excess of deaths in the active treatment group. Finally, 64 (out of 350 planned) patients were recruited to the study.

**Results:**

Sixty-four patients with advanced HF [age—64.2 ± 13.1 years, 57 (89%) men] were enrolled into the study. At baseline visit 34 (53%) patients were randomly assigned to the levosimendan group (study group) and 30 (47%) to the placebo group. Study drug administration resulted in a significant decrease in N-terminal pro-B-type natriuretic peptide concentrations [5084 pg/ml (306–23.203) vs 2027 pg/ml (872–2174), *P* = .02) and left ventricular ejection fraction improvement (20.9 ± 5.9% vs 29.27 ± 5.23%, *P* = .015) in the study group. These patients also had clinical endpoint numerically more often than patients in the placebo group [22 (64.71%) vs 14 (46.67%); *P* = .14], including significantly higher deaths [7 (100%) vs 0, *P* = .02].

**Conclusions:**

In a selected group of stable ambulatory advanced HF (left ventricular ejection fraction ≤35%, New York Heart Association Classes III and IV) patients, repetitive levosimendan 24 h infusion might be an additional therapeutic option but observed deaths may raise its safety issue.

## Graphical Abstract

**Graphical Abstract:**
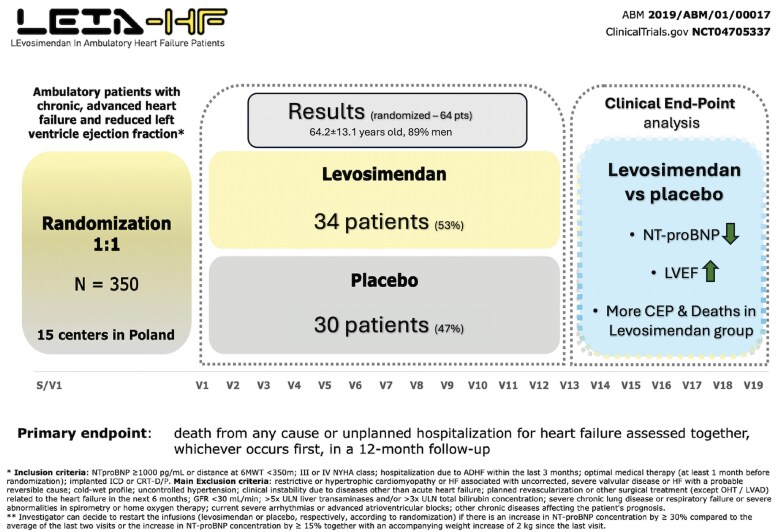

## Introduction

In recent years, advanced heart failure (HF) has emerged as a critical and growing challenge for healthcare systems worldwide.^1,2^ Repeated episodes of cardiac decompensations (ADHF, acute decompensated HF) lead to frequent hospitalizations, worsening prognosis, and increasing treatment complexity. Persistent cardiac dysfunction accompanied by severe symptoms, recurrent pulmonary congestion, episodes of low cardiac output, or malignant arrhythmias contributed significantly to high morbidity and mortality.^2,3^ Notably, every hospitalization due to ADHF is associated with a progressive decline in cardiac function and an increased risk of subsequent rehospitalizations and death.^2,4^ The prevalence of advanced HF is rising, partly due to improved survival following invasive therapies for ischaemic heart disease and the ageing population. This rise presents a clinical burden and imposes substantial financial strain on healthcare systems.^2^

To address acute haemodynamic deterioration in ADHF, inotropic agents such as beta-adrenergic agonists and phosphodiesterase inhibitors (e.g. dobutamine and milrinone) have been commonly used.^5^ While these agents provide short-term improvement in left ventricular (LV) performance and clinical status, large, randomized trials, and meta-analyses have failed to prove long-term mortality benefits.^5,6^ Moreover, concerns about the increased risk of arrhythmias and myocardial ischaemia have limited their extended use.

More than two decades ago, a novel inodilator—levosimendan—was introduced for the treatment of acute HF (AHF).^7,8^ Unlike traditional inotropes, levosimendan provides positive inotropic, vasodilatory, and cardioprotective effects without significantly increasing myocardial oxygen demand. Its mechanism of action involves three complementary pathways: calcium sensitization of cardiac troponin C, which enhances contractility without increasing intracellular Ca^2+^ levels; vasodilation via the opening of ATP-sensitive potassium channels on vascular smooth muscle cells, as well as activation of mitochondrial K^+^-ATP channels in cardiomyocytes, offering protection against ischaemia-reperfusion injury.^8^ These mechanisms enhance cardiac output and stroke volume while also reducing pulmonary artery and capillary wedge pressures. Additionally, levosimendan has shown anti-inflammatory and neurohormonal effects. It lowers proinflammatory cytokines, pro-apoptotic markers such as sFas and Fas-ligand, and suppresses the generation of reactive oxygen species.^9–11^ In patients with HF with reduced ejection fraction (HFrEF), levosimendan has been shown to reduce natriuretic peptide (NP) levels alongside improvements in both systolic and diastolic LV function.^12–14^ Due to its distinct mechanism of action, levosimendan has attracted attention for intermittent, long-term use in patients with advanced HF, including those in palliative care, or awaiting heart transplantation or ventricular assist device (VAD) implantation.

However, despite promising evidence for symptom improvement, NP reduction, better haemodynamics, and fewer hospital readmissions, clinical data on the long-term impact of intermittent levosimendan therapy on survival remain inconclusive. Likewise, other inotropic therapies require cautious use, especially in patients at risk of hypotension and arrhythmias. The study was aimed to determine the safety of repeated infusions of levosimendan in outpatients with advanced systolic HF.

## Methods

### Study design

The study is a subanalysis of levosimendan in ambulatory HF patients (LEIA-HF)—a multicentre, randomized, double-blind, placebo-controlled Phase IV clinical trial (NCT04705337). The study protocol was based on repeated infusions of levosimendan performed in an outpatient setting in patients with end-stage HF. The LEIA-HF study was prematurely terminated at the request of the Data Safety Monitoring Board due to excess of deaths in the active treatment group. For this reason, a detailed analysis of the safety of the tested therapy was performed. The study’s methodology has been described elsewhere.^1^ All patients met following inclusion criteria: age ≥18 years diagnosed with chronic HF with LV ejection fraction (LVEF) ≤35%, in NYHA functional Class III or Ambulatory IV, despite the use of stable, individually optimized pharmacological therapy according to the European Society of Cardiology (ESC) recommendations for at least 1 month before randomization. The study was prematurely terminated due to excess of deaths in the active treatment group. Among 78 pre-screened patients, 64 were randomized—34 to the levosimendan group and 30 to the placebo group. Simple random allocation of individuals between the different intervention groups was done by software randomization tool. Additional inclusion criteria included hospitalization due to HF decompensation within the last 6 months; baseline N-terminal pro-B-type NP (NT-proBNP) concentration ≥1000 pg/ml or distance <350 m in a 6 min walk test (6MWT); additionally, no need for hospitalization at the time of qualification. All subjects were evaluated in a structured manner. A detailed history was conducted regarding major cardiovascular risk factors and the characteristics of pharmacological treatment. A detailed questionnaire was used to collect the medical histories, current health status, socioeconomic and demographic information, and lifestyle habits of all study participants.

Exclusion criteria included hypotension with signs of hypoperfusion [systolic blood pressure (SBP) <80 mm Hg]; uncontrolled hypertension; reversible causes of HF (e.g. alcoholic cardiomyopathy and tachyarrhythmic); advanced renal [estimated glomerular filtration rate (eGFR) <30 ml/min] or hepatic failure; severe lung disease with respiratory failure; paroxysmal heart rhythm disturbances in the last month; pregnancy, postpartum period, or inability to cooperate with the research team.

The primary clinical endpoint (CEP) of the study was composed of death from any cause or unplanned hospitalization for HF, whichever occurred first in a 12-month follow-up period.

All procedures performed in the study were conducted following the ethical standards of the institutional Research Committee (number: APK.002.2.2021) and the 1964 Declaration of Helsinki.

### Randomization and intervention

Patients were randomized to receive either levosimendan (Simdax®, 12.5 mg IV) or placebo. Infusions were administered every 4 weeks, with a mean study duration of 7.6 months. All participants continued optimal medical therapy during the active treatment phase. Each infusion started at a dose of 0.05 μg/kg/min and lasted ∼24 h (up to a maximum dose of 12.5 mg). The intravenous infusion rate was dependent on patient weight and blood pressure values. Before each infusion, patients were assessed for vital signs, electrocardiogram, laboratory tests (including NT-proBNP, lactate, eGFR, and liver function tests) and systolic blood pressure (SBP) measurement on the non-dominant limb. If SBP dropped below 80 mm Hg with signs of hypoperfusion, an algorithm was implemented to reduce the dose or stop the infusion.^1^ Transthoracic echocardiography was performed as described.^1^ The 6MWT was performed during hospital admission and at visits 4, 7, 10, 13, 15, 17, and 19.

### Statistical analysis

The normality of variables was evaluated with the Shapiro–Wilk test. Continuous variables with a normal distribution are typically represented as the mean and standard deviation. Parameters with non-normal distribution are expressed as medians followed by interquartile ranges. Continuous variables with normal distribution were compared with the usage of the *t*-test for independent variables (with the assumption of the equality of variances). Continuous variables with non-normal distribution were analysed with the Mann–Whitney *U* test. Categorical variables were compared using the χ^2^ test. The dependent samples *t*-test or the Wilcoxon signed rank test was used to compare matched (baseline vs follow-up) values, depending on the distribution. *P*-value of <.05 was regarded as statistically significant. All the analyses were performed with STATISTICA, version 11 (StatSoft Inc, Tulsa, Oklahoma, USA).

## Results

### Population characteristics

Sixty-four patients with advanced HF were enrolled in the study: 34 were randomly assigned to the levosimendan group (study group) and 30 to the placebo group (control group). Thirty-two patients (50%) had ischaemic aetiology. The baseline population characteristics are presented in *Table 1*. There were no significant differences between groups regarding age (65.5 ± 12.3 vs 61.4 ± 14.6 years; *P* = .3) or gender (28% vs 24% male; *P* = .9). Haemodynamic parameters, including systolic and diastolic blood pressure and heart rate, also did not differ significantly between the groups. Similarly, there were no significant differences in clinical severity, as defined by ESC guidelines, including NYHA classification and 6MWT results.

**Table 1:** Baseline characteristics of the study group

Patients, *n*	64
Demographics	
Age, years	64.2 ± 13.1
Men, *n* (%)	57 (89)
NYHA class	3.1 ± 0.3
SBP, mm Hg	111.0 ± 13.3
DBP, mm Hg	70.1 ± 9.9
HR, b.p.m.	74.4 ± 14.5
6MWT, m	344 ± 56.6
Medical history	
Diabetes, *n* (%)	30 (46.9)
Hypertension, *n* (%)	48 (75)
Atrial fibrillation, *n* (%)	43 (67.2)
Chronic kidney disease, *n* (%)	30 (46.9)
Prior MI, *n* (%)	26 (40.6)
Prior PCI, *n* (%)	29 (45.3)
Prior CABG, *n* (%)	9 (14.1)
CCS, *n* (%)	32 (50)
PAD, *n* (%)	9 (14.1)
Prior stroke/TIA, *n* (%)	7 (10.9)
Heart failure aetiology	
Ischaemic, *n* (%)	32 (50)
Dilated cardiomyopathy, *n* (%)	19 (30)
Other (myocarditis, valve disease), *n* (%)	13 (20)
Laboratory data	
NT-proBNP, pg/ml	3521 (1670–6628)
Creatinine, mg/dl	1.3 ± 0.43
GFR, ml/min/1.73 m^2^	58.1 ± 19.5
Lactates, mmol/l	2.2 ± 1.1
Sodium, mmol/l	138.4 ± 3.8
Potassium, mmol/l	4.5 ± 0.5
AST, IU/l	27.3 ± 10.7
ALT, IU/l	23.9 ± 16.3
Echocardiography	
LVEF, %	20.8 ± 6.6
Pharmacotherapy	
ARNI, *n* (%)	37 (57.8)
ACEI, *n* (%)	23 (35.9)
BB, *n* (%)	64 (100)
SGLT2i, *n* (%)	61 (95.3)
MRA, *n* (%)	36 (56.3)
LD, *n* (%)	64 (100)
LD dose, mg	58.7 ± 63.9

NYHA, New York Heart Association; SBP, systolic blood pressure; DBP, diastolic blood pressure; HR, heart rate; 6MWT, 6 min walk test; MI, myocardial infarction; PCI, percutaneous coronary intervention; CABG, coronary artery bypass grafting; CCS, chronic coronary syndrome; PAD, peripheral artery disease; NT-proBNP, N-terminal pro-B-type natriuretic peptide; GFR, glomerular filtration rate; AST, alanine transaminase; ALT, aspartate transaminase; LVEF, left ventricle ejection fraction; ARNI, angiotensin receptor-Neprilysin inhibitor; ACEI, angiotensin-converting enzyme inhibitor; BB, beta-blocker; SGLT2i, sodium-glucose cotransporter 2 inhibitors; MRA, mineralocorticoid receptor antagonist; LD, loop diuretics.

### Biochemical characteristics and inflammatory markers

There were no significant differences between these groups in plasma electrolyte levels, serum creatinine concentrations, or eGFR, calculated using the Modification of Diet in Renal Disease formula (MDRD). Despite of the strict adherence to randomization procedures, patients in the levosimendan group had significantly higher baseline plasma NT-proBNP concentrations [5084 pg/ml (306–23.203) vs 2593 pg/ml (133–10.601); *P* = .01] compared with placebo group (*Table 2*). These differences, in turn, had significant impact on results.

**Table 2:** Comparison of levosimendan vs placebo groups at baseline visit

	Levosimendan group *n* = 34	Placebo group *n* = 30	*P*-value
Demographics & clinical assessment			
Age, years	65.5 ± 12.0	61.4 ± 14.6	.3
Men, *n* (%)	28 (82.3)	24 (80.0)	.9
NYHA class	3.0 ± 0.2	3.0 ± 0.0	.8
SBP, mm Hg	111.3 ± 13.1	112.6 ± 13.9	.8
DBP, mm Hg	69.3 ± 11.2	71.2 ± 9.4	.3
HR, b.p.m.	73.2 ± 12.4	71.3 ± 13.7	.8
6MWT, m	388 ± 153	313 ± 124	.2
Laboratory data			
NT-proBNP, pg/ml	5084.0 (306.0–23 203.0)	2593.0 (133.0–10 601.0)	.01
Creatinine, mg/dl	1.3 ± 0.4	1.2 ± 0.3	.7
GFR, ml/min/1.73 m^2^	58.1 ± 19.5	63.7 ± 17.2	.2
Lactates, mmol/l	2.4 ± 1.2	1.9 ± 0.9	.04
Sodium, mmol/l	133.8 ± 3.6	139.1 ± 2.8	.8
Potassium, mmol/l	4.5 ± 0.5	4.5 ± 0.5	.9
AST, IU/l	28.9 ± 13.8	24.4 ± 5.5	.6
ALT, IU/l	27.4 ± 21.1	20.3 ± 7.7	.4
Echocardiography			
LVEF, %	20.9 ± 5.9	22.0 ± 7.4	.5
Pharmacotherapy			
ARNI, *n* (%)	17 (50)	17 (56.7)	.8
ACEI, *n* (%)	14 (41.2)	9 (30)	.3
BB, *n* (%)	34 (100)	30 (100)	1
SGLT2i, *n* (%)	31 (91.2)	30 (100)	.9
MRA, *n* (%)	19 (55.9)	17 (56.7)	.5
LD, *n* (%)	34 (100)	30 (100)	1
LD dose			
Furosemide, mg	58.1 ± 58.3	55.7 ± 72.5	.3
Torasemide, mg	35.5 ± 73.3	22.1 ± 50.8	.2
Endpoints			
Deaths, *n* (%)	7 (20.59)	0 (0)	.02
CEP, *n* (%)	22 (64.71)	14 (46.67)	.14

NYHA, New York Heart Association; SBP, systolic blood pressure; DBP, diastolic blood pressure; HR, heart rate; 6MWT, 6 min walk test; NT-proBNP, N-terminal pro-B-type natriuretic peptide; GFR, glomerular filtration rate; AST, alanine transaminase; ALT, aspartate transaminase; LVEF, left ventricle ejection fraction; ARNI, angiotensin receptor-Neprilysin inhibitor; ACEI, angiotensin-converting enzyme inhibitor; BB, beta-blocker; SGLT2i, sodium-glucose cotransporter 2 inhibitors; MRA, mineralocorticoid receptor antagonist; LD, loop diuretics.

All study patients had serial lactate concentrations assessed to check for signs of hypoperfusion during each visit. Lactate levels did not change significantly (baseline vs Visit 19) during drug infusion in the levosimendan group (2.32 ± 1.19 mg/dl vs 2.24 ± 1.09 mg/dl, *P* = .92). Altogether, 16 patients (47%) had hypotonic episodes during levosimendan infusion, and only 4 episodes required dosage reduction or temporary cessation of the drug infusion. Hypotonia episodes were not associated with CEP or death. Heart rhythm disturbances appeared during levosimendan administration only in six patients—three cases of atrial fibrillation and three cases of non-sustained ventricular tachycardia (nVT).

**Figure 1:**
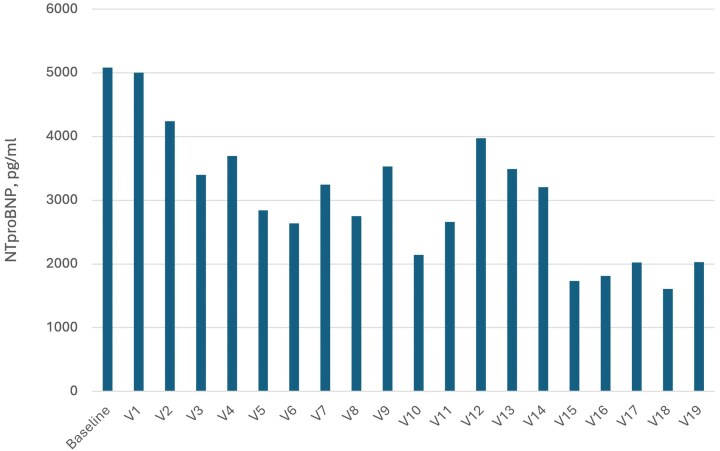
Median N-terminal pro-B-type natriuretic peptide (NT-proBNP) concentrations on every visit in the levosimendan group. A significant decrease was observed between baseline and Visit 19 [5084 pg/ml (306–23.203) vs 2027 pg/ml (872–2174), *P* = .02]

**Figure 2:**
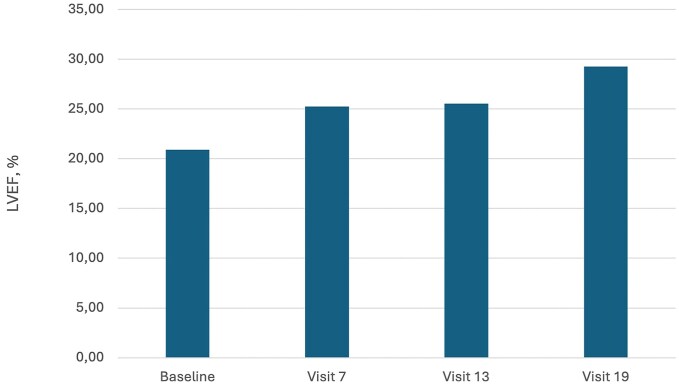
Mean left ventricle ejection fraction (LVEF) values at visits: baseline, 7, 13, and 19 in the levosimendan group. A significant improvement of LVEF was observed between baseline and Visit 19 (20.9 ± 5.9% vs 29.27 ± 5.23%, *P* = .015)

**Figure 3:**
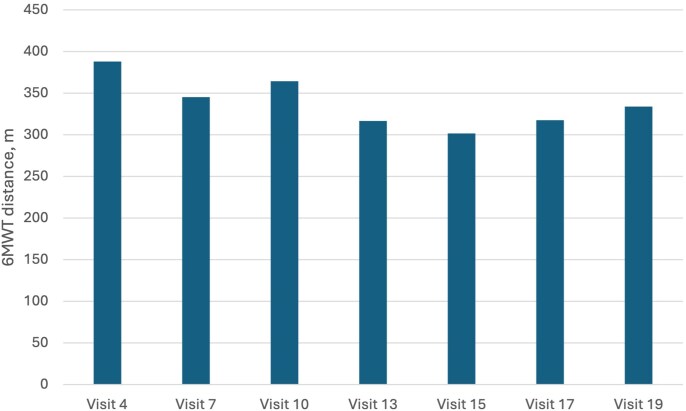
Values of mean 6 min walk test (6MWT) distance at visits: 4, 7, 10, 13, 15, 17, and 19 in the levosimendan group

Importantly, levosimendan administration (baseline vs Visit 19) resulted in a significant decrease of NT-proBNP concentrations [5084 pg/ml (1681–8936) vs 2027pg/ml (872–2174), *P* = .02, *Figure 1*] and LVEF improvement (20.9 ± 5.9% vs 29.27 ± 5.23%, *P* = .015, *Figure 2*). No significant differences were observed in these parameters in placebo group [NT-proBNP 2593 pg/ml (1396–3521) vs 1626 (913–2196), *P* = .51 and LVEF 22.58 ± 5.9% vs 29.4 ± 8.2%, *P* = .27, respectively]. The distance of 6MWTs did not change significantly in both groups (levosimendan group results on each visit are presented on *Figure 3*).

### Clinical endpoint analysis

The mean time to death since baseline visit was 3.14 ± 2.19 months and 2.34 + 0.82 months to hospitalization. There were 34 patients who met the clinical endpoint (CEP) criteria—22 patients (64%) in levosimendan group and 14 (46%) in placebo group. The comparison of both groups is presented in *Table 3*. Interestingly, creatinine concentrations were significantly higher in CEP group (1.4 ± 0.4 mg/dl vs 1.1 ± 0.3 mg/dl; *P* = .003), indicating initially worse renal function. Furthermore, CEP patients requited higher doses of torasemide. These patients also exhibited markedly elevated baseline NT-proBNP levels [3894 (2147–8936) pg/ml vs 1721 (727.8–4164.5) pg/ml; *P* = .001] reflecting greater myocardial stress and disease severity.

**Table 3:** The comparison of patients with and without clinical endpoint (CEP) during observation

	CEP (+) *n* = 36	CEP (−) *n* = 28	*P*-value
Demographics and clinical assessment			
Age, years	63.7 (14.1)	63.4 (12.6)	.8
Men, *n* (%)	31 (86)	26 (92%)	.3
NYHA class	3.0 ± 0.2	3.0 ± 0.0	.8
SBP, mm Hg	110.5 ± 14.5	113.6 ± 11.9	.2
DBP, mm Hg	67.5 ± 8.8	73.6 ± 11.3	.03
HR, b.p.m.	72.2 ± 12.7	71.5 ± 13.7	.8
6MWT, m	391.4 ± 141.4	320.5 ± 138.5	.1
Laboratory data			
NT-proBNP, pg/ml	3894.0 (2147.0–8936.0)	1721.0 (727.8–4164.5)	.001
Creatinine, mg/dl	1.4 ± 0.4	1.1 ± 0.3	.003
GFR, ml/min/1.73 m^2^	54.2 ± 15.4	68.6 ± 19.2	.003
Lactates, mmol/l	2.2 ± 1.2	2.1 ± 1.0	.8
Sodium, mmol/l	138.9 ± 3.4	139.0 ± 3.1	.8
Potassium, mmol/l	4.4 ± 0.6	4.6 ± 0.5	.1
AST, IU/l	27.9 ± 13.1	25.5 ± 7.4	.9
ALT, IU/l	27.1 ± 21.4	20.4 ± 5.9	.8
Echocardiography			
LVEF, %	21.0 ± 6.0	21.9 ± 7.3	.7
Pharmacotherapy			
ARNI, *n* (%)	17 (47.2)	20 (71.4)	.05
ACEI, *n* (%)	14 (38.9)	9 (32.1)	.57
BB, *n* (%)	36 (100)	28 (100)	1
SGLT2i, *n* (%)	35 (97.2)	26 (92.8)	.41
MRA, *n* (%)	18 (50)	18 (64.3)	.25
LD, *n* (%)	36 (100)	28 (100)	1
LD dose			
Furosemide, mg	60.6 ± 60.9	45.7 ± 69.5	.2
Torasemide, mg	40.2 ± 78.9	15.9 ± 37.2	.001

NYHA, New York Heart Association; SBP, systolic blood pressure; DBP, diastolic blood pressure; HR, heart rate; 6MWT, 6 min walk test; NT-proBNP, N-terminal pro-B-type natriuretic peptide; GFR, glomerular filtration rate; AST, alanine transaminase; ALT, aspartate transaminase; LVEF, left ventricle ejection fraction; ARNI, angiotensin receptor-Neprilysin inhibitor; ACEI, angiotensin-converting enzyme inhibitor; BB, beta-blocker; SGLT2i, sodium-glucose cotransporter 2 inhibitors; MRA, mineralocorticoid receptor antagonist; LD, loop diuretic.

In the clinical cohort of 64 patients, seven deaths were recorded (all in the levosimendan group) and two deaths were related to cardiovascular reasons (after unsuccessful left VAD therapy). Patients who died during hospitalization had significantly lower baseline plasma sodium concentrations when compared with survivors (136.0 ± 3.4 mmol/l vs 139.3 ± 3.1 mmol/l; *P* = .02; *Table 4*). Additionally, non-survivors received significantly higher loading doses of torasemide (42.9 ± 39.5 vs 27.3 ± 66.1 mg; *P* = .01), suggesting more aggressive diuretic therapy (*Table 4*).

**Table 4:** The comparison of deaths vs survivors

	Deaths *n* = 7	Survivors *n* = 57	*P*-value
Demographics and clinical assessment			
Age, years	63.1 ± 9.2	63.6 ± 13.8	.6
Men, *n* (%)	6 (85.7)	51 (89)	.9
NYHA class	3.0 ± 0.0	3.0 ± 0.1	.9
SBP, mm Hg	108.4 ± 16.5	112.4 ± 13.0	.3
DBP, mm Hg	68.4 ± 4.9	70.5 ± 10.9	.7
HR, b.p.m.	71.2 ± 9.6	74.4 ± 8.8	.7
6MWT, m	476.7 ± 37.9	333.5 ± 141.8	.04
Laboratory data			
NT-proBNP, pg/ml	7008.0 (1670.0–23 203.0)	2798.0 (1551.0–5260.0)	.06
Creatinine, mg/dl	1.4 ± 0.4	1.2 ± 0.4	.2
GFR, ml/min/1.73 m^2^	50.8 ± 12.1	61.9 ± 18.9	.1
Lactates, mmol/l	2.5 ± 1.3	2.2 ± 1.1	.5
Sodium, mmol/l	136.4 ± 3.4	139.3 ± 3.1	.02
Potassium, mmol/l	4.3 ± 0.5	4.5 ± 0.5	.5
AST, IU/l	35.9 ± 17.4	25.7 ± 9.4	.2
ALT, IU/l	35.4 ± 30.7	22.7 ± 13.8	.5
Echocardiography			
LVEF, %	21.4 ± 5.9	21.4 ± 6.8	1.0
Pharmacotherapy			
ARNI, *n* (%)	3 (42)	31 (54)	.56
ACEI, *n* (%)	4 (57)	19 (33)	.76
BB, *n* (%)	7 (100)	57 (100)	1
SGLT2i, *n* (%)	5 (71)	56 (86)	.01
MRA, *n* (%)	4 (57)	32 (56)	.95
LD, *n* (%)	7 (100)	57 (100)	1
LD dose			
Furosemide, mg	62.9 (89.0)	56.2 (62.0)	.7
Torasemide, mg	42.9 (39.5)	27.3 (66.1)	.01
Endpoints			
Deaths, *n*	7	0	
CEP, *n* (%)	7 (100)	14 (24.5)	.16

NYHA, New York Heart Association; SBP, systolic blood pressure; DBP, diastolic blood pressure; HR, heart rate; 6MWT, 6 min walk test; NT-proBNP, N-terminal pro-B-type natriuretic peptide; GFR, glomerular filtration rate; AST, alanine transaminase; ALT, aspartate transaminase; LVEF, left ventricle ejection fraction; ARNI, angiotensin receptor-Neprilysin inhibitor; ACEI, angiotensin-converting enzyme inhibitor; BB, beta-blocker; SGLT2i, sodium-glucose cotransporter 2 inhibitor; MRA, mineralocorticoid receptor antagonist; LD, loop diuretics; CEP, clinical endpoint.

## Discussion

Although the positive effects of levosimendan on haemodynamics, symptoms, and biomarker concentrations have been relatively consistent across many studies, its influence on mortality remains a matter of debate.^15–18^ The results of the LEIA trial did not prove a favourable effect of levosimendan on the prognosis of patients with advanced HF. Due to the finding of excess of deaths in patients receiving the drug, although the difference was not statistically significant, it requires detailed analysis and scientific reflection. The results in terms of mortality from other studies also remain inconclusive.

In a randomized, double-blind LIDO trial, conducted in patients with ADHF, levosimendan outperformed dobutamine in improving haemodynamic parameters and reducing 180 day mortality.^15^ Its mechanism of action included enhanced cardiac output without increasing myocardial oxygen demand. Next, the RUSSLAN study evaluated the safety and efficacy of levosimendan in a different clinical setting—patients with AHF following acute myocardial infarction.^16^ In that trial, 504 patients received relatively high levosimendan doses (0.1–0.4 μg/kg/min) or placebo over a 6 h infusion. The authors concluded that levosimendan significantly reduced the risk of death or worsening HF during infusion and at 24 h. Mortality was also lower at 14 days, with a sustained trend at 180 days.^16^

However, the above results were not replicated in other studies concerning ADHF patients.^17,18^ In the REVIVE trial, a 24 h infusion of levosimendan resulted in fewer episodes of HF decompensation, shorter hospital stays, and symptomatic improvement. However, these benefits lasted only 5 days, and by Day 90, the risk of death was numerically higher in the treatment group than in the placebo group, particularly in patients with lower baseline SBP values.^17^ Similarly, the SURVIVE trial did not demonstrate a mortality benefit. Despite a greater reduction in BNP levels in the levosimendan group during the first days, all-cause mortality at 180 days was comparable to the group treated with dobutamine.^18^

Our study aimed to assess whether levosimendan benefits patients with advanced HF and investigate whether baseline clinical characteristics influenced the response to levosimendan. Notably, our treatment protocol and follow-up periods were longer than in previous trials, allowing for more comprehensive evaluation of levosimendan therapeutic potential in this population.

Repetitive administration of the drug resulted in a significant decrease in NT-proBNP concentrations and an increase in LVEF. Notably, the effect on NT-proBNP concentration was limited to the treatment phase and the first visit after treatment termination. Similar to previous studies, our results suggest that levosimendan exerts a heart-protective action, probably through neurohormonal and anti-inflammatory effects. This, in turn, results in improved haemodynamics—an increased LVEF and reduced NT-proBNP levels. However, this effect may be limited only to the period of treatment. The NT-proBNP concentration after the follow-up phase did not differ significantly from baseline values. In the LevoREP trial, NT-proBNP concentration was also significantly lower after 8 weeks of treatment compared with baseline values, but not after 24 weeks.^19^ A significant decrease in NT-proBNP concentration was documented in the LION-HEART study after 12 weeks, directly following treatment.^20^ It suggests that levosimendan and its active metabolites have a positive effect on heart haemodynamics as long as infusions are repeated systematically. In our study, the impact on ejection fraction remained positive even on the last follow-up visit. It is the only trial assessing LVEF 6 months after termination of treatment. So far, LVEF improvement was documented 7 days^21^ or a month^22^ after levosimendan infusion. Our study did not find statistically significant changes in the 6MWT distance, consistent with the findings of the LeoDOR and LevoREP trials.^19,23^

In the LEIA-HF study group, we observed seven all-cause deaths and 36 patients reached CEP. Likewise, in the LeoDOR trial,^23^ the levosimendan group presented with a higher incidence in components of the primary endpoint, including death, VAD implantation or high-urgent heart transplant (treatment lasted for 12 weeks). Moreover, the highest event rate was observed among patients with the highest cumulative levosimendan dose (higher for 24 than for 6 h infusion). On the contrary, LevoREP study showed significant reduction in the risk of death, heart transplantation, or AHF after 24 weeks (6 weeks of treatment and 18 weeks of follow-up),^19^ whereas LION-HEART showed significant reduction in the rate of HF hospitalizations in levosimendan patients compared with placebo (3 months of treatment and 9 months of observation).^20^ In the LAICA study, levosimendan treatment was administered for 12 months and reduced the incidence of HF decompensation episodes and death only at 1 and 3 months, whereas the results at months 6 and 12 were nonsignificant.^24^

The inconclusive results concerning mortality after levosimendan treatment in chronic advanced HF are probably caused by heterogeneous trial protocols. Different studies implemented different doses of levosimendan, randomized patients according to different inclusion and exclusion criteria, and assessed them at various time points.

The LevoREP study recruited patients with significantly lower NT-proBNP concentration, excluded patients with SBP < 100 mm Hg (LEIA-HF < 85 mm Hg) and withheld diuretic administration on the day of levosimendan infusion.^19^ Patients recruited to the Lion-HEART study received less intensive HF treatment, as only ∼70% received beta-blockers, angiotensin-converting enzyme inhibitors (ACEIs), or mineralocorticoid receptor antagonists, and nobody in either the LION-HEART or the aforementioned trials received angiotensin receptor-neprilysin inhibitors (ARNIs).^20^ Furthermore, in both studies, levosimendan infusions lasted only 6 h, and the treatment was terminated after 6 weeks in LevoREP^19^ and 12 weeks in LION-HEART.^20^ These specific protocols, including better clinical and haemodynamic state of recruited patients, less intensive background HF therapy, and lower doses of levosimendan, may have resulted in better outcomes for treatment groups in both studies.

LEIA-HF recruited subjects with the lowest mean ejection fraction compared with other studies. All patients received complex background HF therapy, with 100% of patients treated with a beta-blocker, 35.9% receiving ACEI, and 57.8% receiving ARNI. Furthermore, levosimendan infusions lasted 24 h at a dose of 0.05 μg/kg/min.

Similarly, 24 h infusions were implemented in the LeoDOR study.^23^ Recruited patients also presented with a high intensity of guideline-directed medical treatment, with >90% of patients treated with beta-blockers and ACEI. LeoDOR authors concluded that combining high-dose inodilators with neurohumoral antagonists might not have a favourable effect on peripheral perfusion and, thus, the event rate. This might also explain our study results.

It was already known that a combination of levosimendan with hypotensive drugs administered in HF often results in hypotension leading to ischaemia and reduced vital organ perfusion.^17,25^ Myocardial ischaemia during hypoperfusion is particularly considered in patients with ischaemic HF aetiology. However, in the LEIA-HF study, episodes of hypotension were not associated with CEP occurrence or death. Moreover, lactate levels did not change significantly during drug infusion compared with the screening visit in the levosimendan group. Furthermore, CEP patients presented with different HF aetiologies, which is essential considering low-pressure values.

In the LEIA-HF study, patients who died had lower baseline serum sodium concentration and needed higher doses of torasemide. Next, patients with CEP presented with higher NT-proBNP concentrations, lower diastolic pressures, higher creatinine concentrations, and required higher doses of torasemide. Moreover, CEP patients randomized to levosimendan presented with even higher NT-proBNP values and higher lactate levels. These findings suggest that an adverse prognosis might be associated with an initial state of left ventricle overload, signs of congestion and decreased perfusion of non-cardiac organs, including kidneys. It is possible, that best benefits from levosimendan treatment would be achieved in early phase of HF decompensation. Visco et al. evaluated the effects of levosimendan administration in individuals with first signs of haemodynamic congestion detected with implantable CardioMEMS device. Patients with increasing pulmonary artery pressures despite maximal changes in HF therapy were hospitalized for levosimendan infusion. The strategy resulted in reduced length of hospital stay, improved quality of life, and lower costs.^26^

Dobarro et al.^27^ attempted to develop an algorithm (LEVO-D score) to predict a positive response to levosimendan treatment. Among the parameters predicting a better prognosis were lower NT-proBNP concentration, lower heart rate, beta-blocker treatment, higher haemoglobin level, previous transcatheter edge-to-edge repair, and no HF visits in the last year. In responders, the authors observed a significant reduction in HF admissions, unplanned HF visits, as well as the reduced incidence of CEP during the year after treatment.^26^

Considering these facts, it should be emphasized that despite strict adherence to randomization procedures in the LEIA-HF study, the results were biased of baseline differences between study groups. It can be assumed with high probability that continuation of the study, allowing for an increase in the size of the study population, would have led to a balance of risk between the groups, better reflecting the real impact of levosimendan on the clinical outcome.

It should therefore be assumed that the low number of enrolled patients is the main limitation of the LEIA-HF study, which paradoxically led to its premature termination.

In general, levosimendan represents a promising therapeutic option in the management of ADHF, particularly for symptom relief and bridging strategies. In a selected group of patients with advanced HFrEF (especially with high concentrations of NT-proBNP), levosimendan 24 h infusion might be the only option to receive diuresis and decongestion, which resulted in discharge. Concluding, further well-powered, randomized controlled trials are needed to conclusively determine its effect on long-term outcomes and mortality in patients with advanced HF. Moreover, the drug dosing regimen should also be revised and tailored to the patient, as levosimendan exerts a positive haemodynamic effect only within a limited time.

## Conclusion

To conclude, presented results may suggest that only patients with specific clinical phenotypes of advanced HF would benefit from the intermittent levosimendan treatment. It is challenging to distinguish these patients using simple criteria, including symptoms, LVEF, blood pressure values, NYHA class, or NT-proBNP concentration. At some point in the disease's progression, the administration of levosimendan may not be beneficial in terms of clinical outcomes—in presented study deaths occurred significantly more frequently in the active arm and exclusively in the levosimendan group. This may raise its safety issue for potential candidates.

### Study limitations

The main limitation of the study is baseline difference between the study and placebo group considering NPs concentrations. This may reflect initially worse clinical condition of subjects recruited to levosimendan administration. Indeed, deaths occurred only in levosimendan group what was challenging for further statistical analysis. Furthermore, the study was prematurely discontinued; therefore, lower number of participants (as expected) was enrolled.

## Data Availability

The data underlying this article will be shared on reasonable request to the corresponding author.
